# As time passes by: Observed motion-speed and psychological time during video playback

**DOI:** 10.1371/journal.pone.0177855

**Published:** 2017-06-14

**Authors:** Thomas Jonathan Nyman, Eric Per Anders Karlsson, Jan Antfolk

**Affiliations:** Department of Psychology, Faculty of Arts, Psychology and Theology, Åbo Akademi University, Turku, Finland; Rijksuniversiteit Groningen, NETHERLANDS

## Abstract

Research shows that psychological time (i.e., the subjective experience and assessment of the passage of time) is malleable and that the central nervous system re-calibrates temporal information in accordance with situational factors so that psychological time flows slower or faster. Observed motion-speed (e.g., the visual perception of a rolling ball) is an important situational factor which influences the production of time estimates. The present study examines previous findings showing that observed slow and fast motion-speed during video playback respectively results in over- and underproductions of intervals of time. Here, we investigated through three separate experiments: a) the main effect of observed motion-speed during video playback on a time production task and b) the interactive effect of the frame rate (frames per second; fps) and motion-speed during video playback on a time production task. No main effect of video playback-speed or interactive effect between video playback-speed and frame rate was found on time production.

## Introduction

Psychological time is a fascinating and poorly understood evolutionary adaptation and denotes the subjective experience and assessment of the passage of time. Contemporary research suggests that psychological time is associated with many aspects of human cognition (both within and across sense modalities), action (one’s own and other’s), and emotion [1–7]. It has been shown that psychological time is malleable and that the central nervous system (CNS) re-calibrates information in accordance with internal and external factors so as to flow slower or faster [1, 3, 8]. In other words, there seems to be a flexible temporal system within the CNS which adapts to situational influences. Moreover, there are many models describing how the CNS might manage and measure the rate of psychological time; ranging from neurologically based descriptions of an ‘internal clock’ to purely cognitive descriptions, but there is as yet no clear consensus regarding how the mechanisms within the CNS assess temporal information [9–13].

An important situational factor which has been shown to influence subjective time is observed motion [3, 14–17]. It has, for example, been found that the psychological experience of duration lengthens, or dilates, when observing a stimulus that is moving (e.g., a rolling ball) compared with observing a stimulus that is stationary or moving more slowly [15–17]. The aim of the present study was to examine the connection between perceived motion and time production. This was achieved by studying the effect of motion speed on subjective time through a supra-second (i.e., more than one second long) production task where individuals were asked to continuously produce intervals of time by pressing a keyboard key every a nine seconds (see Experiment 1: Method).

### Motion and time estimation

It has been shown that time production (i.e., producing intervals of time) and estimation (i.e., assessing time intervals) is affected by both actual motion and implied motion; an example of implied motion is a static image of an object which only *appears* to be in motion [18]. Furthermore, the importance of motion with regard to time estimation and the production of time estimates can perhaps best be understood from an evolutionary perspective. From such a perspective it has been assumed that selective pressures during our natural history has given rise to a temporal system that takes into account the flow of the external world and readjusts accordingly [3, 14]. For example, the human ability to estimate the distance of an oncoming threat and the time it will take to run away from or be caught up by that threat. In other words, the prediction and timing of coming events requires an assessment of both space and time.

An investigation of the above mentioned association between motion-speed and time production can be found in a conference abstract by Eagleman [14], the full study of which has not been published. In this abstract the author reports an investigation of the effects of motion-speed on a duration judgment task. Here the manipulation of motion-speed was achieved with the help of slow and normal video playback-speed of a movie-sequence depicting natural biological motion. In other words, video playback-speed of motion was treated as equal to motion-speed because increased or decreased video playback depicting motion resulted in increased and decreased motion-speed. Eagleman [14] concluded that slow-motion video playback resulted in the judgement of a flash to be shorter in duration compared with a flash with the same duration during normal video playback. Furthermore, no such differences in duration judgments were found when the frames and pixels of the video sequences were scrambled. The results were interpreted as a demonstration of the central nervous system’s ability to recalibrate the inner flow of time in accordance with the perceived motion-speed in the external environment so as to be able to make spatiotemporal predictions with regard to the observed motion. Moreover, in a study by Grivel, Bernasconi, Manuel, Murray, and Spierer [19], video playback-speed was used to investigate the effect of motion on time production. Here, slow-, normal-, and fast playback of a video sequence depicting people walking across a square was used to investigate the effect the different video playback speeds had on time production. The investigators found that the production of time intervals was influenced by the speed of video playback so that fast speed resulted in underproductions of intervals of time compared with slow speed, which resulted in overproductions of time. The difference between the two mentioned studies is the task utilized, the former being a duration judgement task and the latter being a production task. It should however be noted that both used prospective designs (i.e., the participants knew beforehand that they would be estimating time) [1, 20–22]. In sum, both studies suggest that psychological time is influenced by the observed motion-speed during video playback.

### Observed motion as distinguishable events

An additional association between psychological time and observed motion is evident in the view put forward by Fraisse [23], who suggested that motion should be understood as the noticeable changes between distinguishable events. In other words, the perception of motion is the result of seeing a succession of different events (e.g., seeing a ball that rolls from one side of a room to another can be understood as seeing a sequential display of multiple images representing the ball’s trajectory). Fraisse [23] also stated that increasing the number of observed distinguishable changes dilates the perceived passage of time and viewed this as an indication that the CNS interprets more changes in events as more time having passed. In accordance with this view, one might posit that a higher density of successive events increases the perceived duration of the passage of time because the CNS gauges time in accordance with the amount of changes observed [16, 24]. According to Fraisse’s interpretation, one could infer that the effect found in previous studies between video playback-speed and time production is due to the perception of changes in events. That is, fast playback is perhaps perceived as including more event changes, whereas slow playback is perhaps perceived as including less event changes.

A further indication of the association between event changes and the perception of motion is a study by Kanai, Paffen, Hogendoorn, and Verstraten [25] in which the authors explored, among other aspects, the association between event changes and time estimation. They found that a higher frequency of flickering of a stimulus influenced time estimation so that time estimates were dilated. They interpreted their results as showing that it is temporal frequency rather than motion-speed which influences time estimation. However, Kaneko and Murakami [26] later contested these results by providing support for the notion that it is indeed the motion-speed that indexes time and not simply temporal frequency. Lastly, in a study by van Rijn [27] it was shown that there is a link between observed visual changes and temporal dilation. The experiment entailed observing, from a first-person perspective, a car in high speed motion and making judgements of the duration of the observed sequence. The results showed that the increased observed speed increased the number of observed contextual changes, and van Rijn [27] concluded that visual changes are associated with temporal processing. In sum, research suggests that there is an association between psychological time and the observed motion-speed of events and that the perception of motion and time are perhaps also linked to the number of perceived changes of events.

### Motion and frame rates in film

Considering the above raised aspects, one way of further exploring the association between time production and observed motion would be to investigate how noticeable changes of events and motion-speed interactively affect the production of intervals of time. Such an exploration might be compared with how a more fluid and less segmented observable motion influences time production. A novel way of conducting such an investigation would be to explore how higher versus lower frame rates (frames per second: fps) of video sequences depicting observable motion affect time production. In the context of motion picture, the term “frames per second” refers to the rapidity with which images are either successively captured or displayed [28]. In cinematographic movies the conventional recording and displaying rates are 24 fps or 25 fps. For the purposes of this study, and for the sake of clarity, the term “frames per second” will only be used to denote the speed at which film has been recorded and the terms “playback speed” and “motion speed” will be used to denote the speed at which the film is displayed. In this context, the terms “playback speed” and “motion speed” are interchangeable due to the fact that motion speed is the same as the speed at which the video sequence is displayed. The reason for focusing on both the number of frames used to record a video sequence (fps) and the rate at which it is displayed (i.e., playback speed or motion speed) is because it is known that higher fps will lead to a smoother visual perception of motion compared with lower fps, which will lead to a more uneven visual perception of motion [29]. To the best of the authors’ knowledge there have been no studies which have expanded on the reported effects of video playback-speed on time production. Nor has there been any examination of the interactive effect between fps used to record a video sequence and the video playback speed on time production.

### The current study

The aims of the current study were to experimentally investigate: 1) the main effect of observed motion-speed during video playback on time production and 2) the interactive effect of fps and motion-speed on time production. The first aim was to conceptually replicate previous findings [14, 19] and the second was to further investigate if the effects of video playback speed on time production is caused by the perceptual difference of event changes between slower and faster playback. In order to achieve these goals, three experiments were designed and conducted. Experiment 1 (*n* = 28) included three conditions of motion-speed, that is, slow playback (80%), normal playback (100%), and fast playback (120%). Experiment 2 (*n* = 57) was comprised of the same three conditions of motion-speed presented with two levels of fps (25 fps and 50 fps). Experiment 3 (*n* = 29) included a baseline measure and three conditions of motion-speed (i.e., slow playback; 80%, normal playback; 100%, and fast playback; 120%). Taken together, these experiments enabled us to estimate, and independently replicate, a general effect of motion-speed on the production of intervals of time. More specifically, the produced intervals of time were compared between conditions in order to ascertain if the subjective interval productions were over- or underproductions. The hypotheses of Experiment 1 and 3 were that slow and fast video playback-speeds would result in over- and underproductions of time, respectively. The postulates of Experiment 2 were the same as Experiment 1and 3 but with the additional hypothesis that fps and video playback-speed would have an interactive effect on time production. It was hypothesized that the interactive effect would result in low fps giving rise to a more uneven visual perception in all conditions of video playback (i.e., slow, normal, and fast) compared with high fps in all conditions. The uneven visual perception of motion was believed to result in more noticeable changes of events and, therefore, to be interpreted by the CNS as more time having passed. The expectation was that the uneven rate of motion would result in clearer underproduction of time.

## Experiment 1

The aim of the first experiment was to investigate, as a conceptual replication of previous evidence [19], how three conditions of motion-speed during video playback (i.e., slow(80%), normal(100%), and fast(120%)), depicting observable motion, influence the production of nine-second intervals of time. The hypotheses were based on previous findings [19] and were: a) that slow playback-speed would result in overproductions of nine-second intervals compared with the normal condition and b) that fast playback-speed would result in underproductions of nine-second intervals compared with the normal condition.

### Method

#### Participants

A convenience sample of 30 Swedish-speaking Finnish students (including 24 women and 6 men; age range 20 to 30) at Åbo Akademi University participated in the experiment. Participants were recruited via e-mail or were asked to join while visiting the university canteen. Participants were randomized into three separate experimental conditions (see Experiment 1: Procedure). All participants gave their informed consent before taking part in the experiment. After completion, all participants received one cinema ticket each.

#### Ethics statement

The current study, including Experiments 1, 2 and 3, was approved by the Ethical Committee at the Department of Psychology and Logopedics at Åbo Akademi University.

#### Measures

Prior to the experiment, three self-assessment forms were administered in order to investigate factors previously shown to influence timing tasks (e.g., tiredness, anxiety, and psychopathology) [7]. Psychopathology and psychological symptoms were measured using a Swedish translation of the Symptom Checklist 90 (SCL 90) [30, 31], which includes the following variables: Global Severity Index, Positive Symptom Distress Index, Phobic Anxiety, Obsessive-Compulsive, Paranoid Ideation, Psychoticism, Interpersonal Sensitivity, Depression, Anxiety, Hostility/Anger, and Somatization.

Levels of state anxiety were assessed using 20 items from the State Trait Anxiety Inventory (STAI), which had been translated into Swedish from English [32]. The 20 items were taken from the State-scale of the questionnaire and were divided into two sets of 10 items. Each participant answered 10 pre-test questions and 10 post-test questions, which resulted in three variables: 1) the pre-test scores (10 questions), 2) the post-test scores (10 questions), 3) and the pre- and post-test scores combined (20 questions).

Furthermore, time orientation was measured using a Swedish translation of the Zimbardo Time Perspective Inventory (S-ZTPI) [33, 34]. Time orientation was included in order to investigate differences in the production of time intervals depending on how individuals relate to the past, the present, and the future. The inventory used in the first experiment was based on the original five variables used by Zimbardo and Boyd [34]: Past Positive Orientation, Past Negative Orientation, Present Fatalistic Orientation, Present Hedonistic Orientation, and Future Orientation.

#### Stimulus

The stimulus used in Experiment 1 was a video sequence that was captured with a Sony Handycam (DCR-PC 350 E), which uses 25 fps as the standard recording and playback-speed. The original video was approximately 20 minutes in length and was recorded from the second floor of an indoor market space (i.e., the sequence was captured from above and the camera angle was tilted slightly downwards). The video resolution was 320 x 240 pixels and the video sequence depicted people walking back and forth through an indoor market place a circumscribed space. The stimuli were presented without audio on a 2.8 GHz iMac computer with a 24-inch LED-monitor and controlled by the software Presentation (version 12.1.) The display resolution for the video playback was 800 x 1280 pixels; a result of increasing the size of the original video (i.e., 320 x 240 pixels) in order to facilitate a greater visual experience of the recorded video.

Due to the difference in motion-speed in the three conditions, that is, slow (80%), normal (100%), and fast (120%) and because a fixed 12 minute duration was chosen for the presented video sequences, there was also a difference in the number of frames viewed throughout the 12 minutes of video playback in each condition. That is, there was a difference in the overall content due to the different playback-speeds. This meant that although all conditions included a 12-minute video sequence, the slow condition contained a total of 14400 frames, the normal condition contained a total of 18000 frames, and the fast condition contained a total of 21600 frames. This difference in total number of frames was achieved by editing the original 20-minute video to fit the three conditions. In other words, the content of each movie was equal in duration but unequal in the amount of recorded footage shown; those watching the video sequence with more frames saw more of the uniform movement than those watching the sequence with fewer frames. This is a difference in content, unlike the difference in the number of frames used to represent the same content; which was the aim of the manipulation of the frames in the second experiment (see Experiment 2: Measures). The authors’ do not know of any previous research on time production in which a video sequence of such a long duration (i.e., 12 minutes) has been used. We acknowledge that the choice of this duration was arbitrary.

#### Interval duration

The exact length of the target interval of time for the production task (i.e., nine seconds) was based on previous research indicating important duration boundaries when studying interval timing [1, 35]. One important distinction is made between sub-second and supra-second durations. The former is of interest when investigating neurophysiological mechanisms and the latter in the case of cognitive mechanisms, such as working memory and attention [1]. Another distinction, made by Fraisse [36], is between the perception of duration (approximately below three seconds) and the estimation of durations (approximately above three seconds); which has also received support in later research [37]. Fraisse [36] argued that shorter durations (i.e., below three seconds and in some cases even below five seconds) might be experienced as single units and not as separable events in time. In the present experiment, the target nine-second interval (i.e., a supra-second duration) was chosen because 1) it represented a duration that was clearly higher than the three and five seconds and 2) engaged, in accordance with Fraisse [36] and Grondin [1], the cognitive mechanisms involved in the estimation of durations of time. Interestingly, Grivel et al., [19] chose a target interval of 20 seconds, which is potentially problematic as it has been found that there is a linear relationship between duration and estimation error [2, 38]. It was, therefore, deemed rational to keep the target interval duration of the present experiments as low as possible but still clearly above the three to five- second threshold mentioned by Fraisse [36]. To the authors’ knowledge there is no previous research in which a nine-second target interval in a production task has been used. We acknowledge that the choice of this duration was arbitrary.

#### Procedure

A pilot experiment with a within-subject design revealed that the main effect of motion-speed during video playback was possibly masked by a strong order effect (see Pilot Experiment in S1 File); therefore, a between-individual design was employed in the present experiment. Three separate experimental conditions were created. The first condition was a slow video playback (80% playback-speed), the second was a normal video playback (100% playback-speed), and the third condition was a fast video playback (120% playback-speed). In each of the three conditions, 10 participants were instructed to view a video sequence lasting 12 minutes. The task in each condition was to observe a 12-minute low-resolution video sequence and continuously produce nine-second target intervals. The task was conducted in a secluded, low stimulus room wearing earplugs and headphones that muted external sounds. The production of time intervals was achieved by pressing a key on a keyboard every time the participant perceived that nine seconds had elapsed since the last key press.

Participants were not asked to avoid timing strategies, such as chronometric counting. The reason for this were twofold: Firstly, timing strategies have been shown to be notoriously difficult to avoid unless additional tasks are included to block out the possibility of counting or asking participants not to count [39]. Secondly, the results from the pilot experiment did not support the notion that there would be a difference if the participants were asked not to count (see Pilot Experiment in S1 File).

### Statistical analyses

Before the main analysis was conducted, 133 outliers were identified and removed. Prior to removal of outliers there was a total of 2332 produced time estimates and after there was a total of 2199 produced time estimates. This was accomplished by the use of standardized residuals (z-scores), with a cut off margin of 95% [-1.96 and 1.96], based on the entire sample. This was a precaution used in order to decrease the risk of measurement error due to inattentiveness and boredom during the experiment (e.g., forgetting to press every nine seconds and instead pressing after a long delay). A further step, which was taken before the main analysis was conducted, was a clustering of the data. The data was clustered so that three mean time productions were calculated per individual. These three time productions corresponded to 1) the beginning (estimate 1; the mean value of all estimates between 0–240 seconds from the start of the experiment), 2) the middle (estimate 2; the mean value of all estimates between 241–480 seconds), and 3) the end of the video (estimate 3; the mean value of all estimates between 481–720 seconds). This method was used considering that the total number of observations (i.e., produced target intervals of time) and the points in time for each observation varied between individuals (an inherent aspect of individuals producing continuous time intervals). After clustering, investigation showed that the dataset was normally distributed on each level of the independent variable. A boxplot analysis revealed two additional outliers in the clustered data distributions (in the first and fourth interquartile groups), which required the removal of two participants from the fast condition. Subsequent to screening, 28 participants were included in main analysis; 10 participants in the slow condition, 10 participants in the normal condition, and 8 participants in the fast condition.

After the clustering of the data the dataset was analyzed using repeated measures ANOVA with time estimation as a within-subjects factor (estimate 1, estimate 2, and estimate 3) and condition as a between-subjects factor (slow, normal, fast).

All background variables (i.e., the results from the STAI, the SCL-90, and the S-ZTPI) were correlated, using Pearson correlation (two-tailed), with the average time productions of all participants in order to investigate whether or not there were any associations between the background data and the outcome data.

### Results

**Fig 1:**
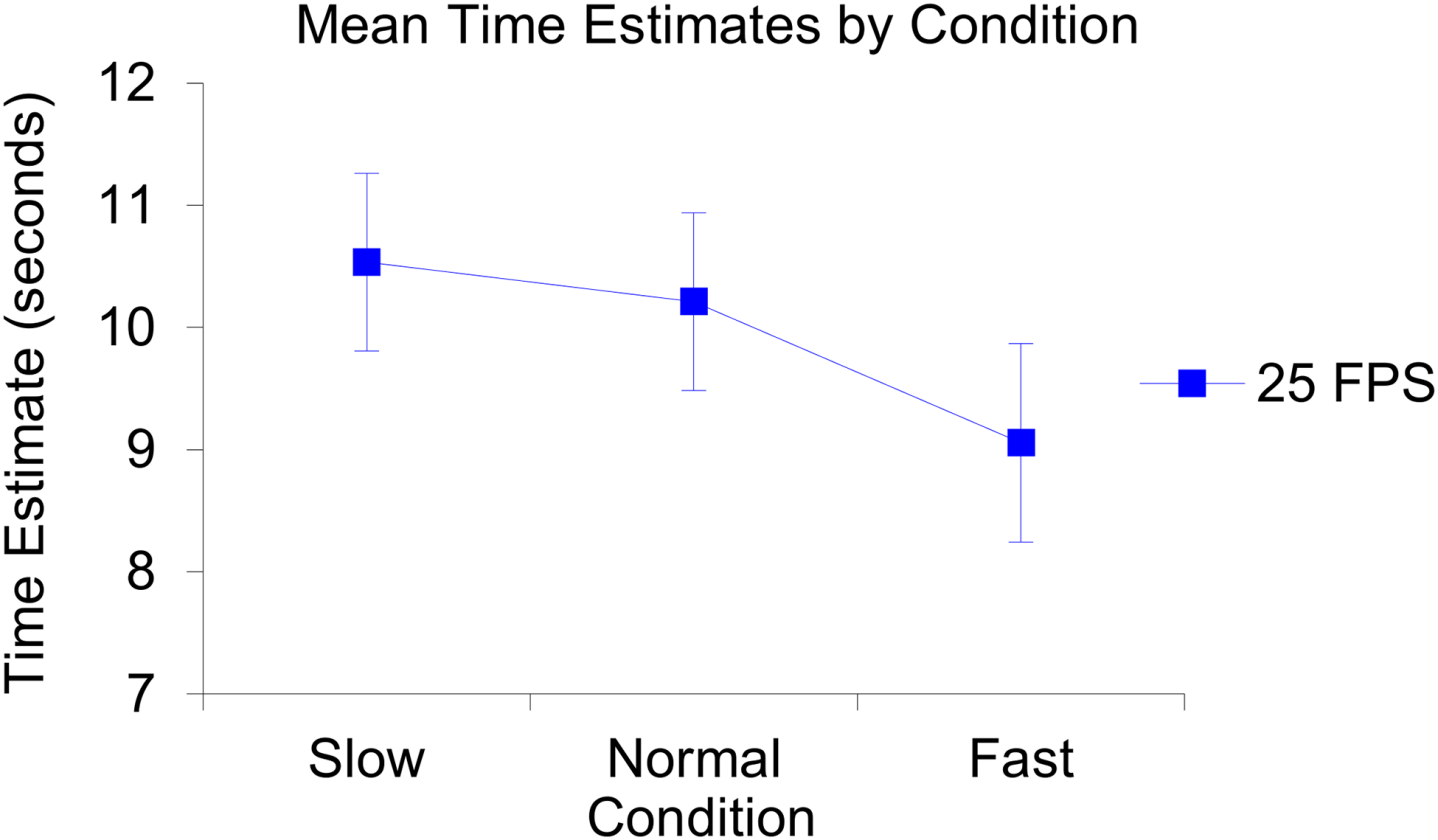
Illustration of the produced mean time estimates per condition. The slow condition (*n* = 10, *M* = 10.54, *SE* = 0.73), the normal condition *(n* = 10, *M* = 10.21, *SE* = 0.73) and the fast condition *(n* = 8, *M* = 9.06, *SE* = 0.81). The error bars represent the standard errors of the mean in each condition.

**Fig 2:**
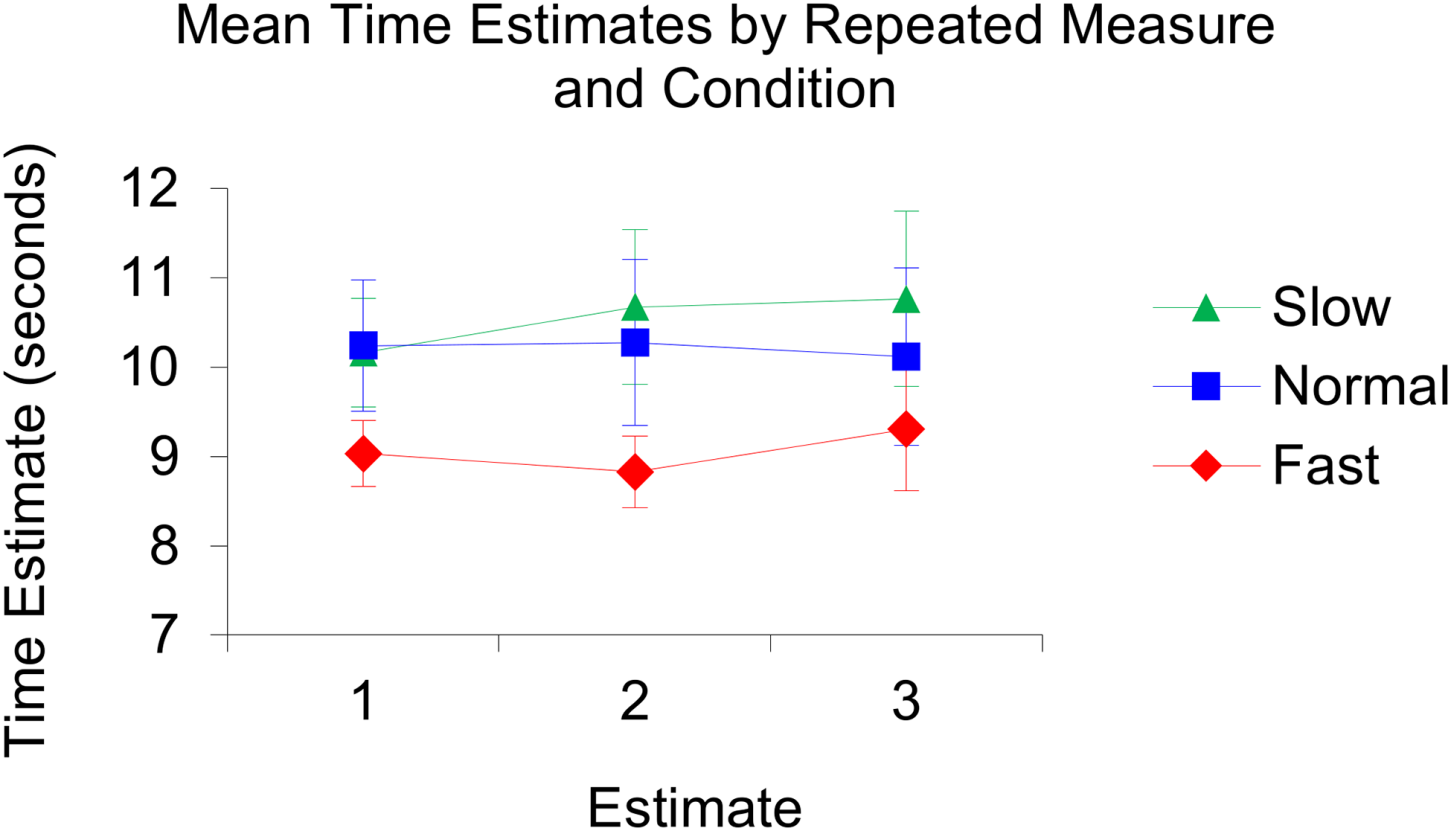
Illustration of the produced mean time estimates per repeated measure and per condition. In the slow condition: Estimate 1; 10.16, estimate 2; 10.68, estimate 3; 10.77. In the normal condition: Estimate 1; 10.24, estimate 2; 10.27, estimate 3; 10.12. In the fast condition: Estimate 1; 9.04, estimate 2; 8.82, estimate 3; 9.30.

The repeated measures ANOVA revealed that the Mauchly’s test of sphericity had been violated, (χ2(2) = 19.66, *p* < .0001), and therefore the degrees of freedom were corrected for by using the Greenhouse-Geisser estimates of sphericity, (ε = 0.64). There was no significant difference between the three repeated measures (i.e., the three time clusters), *F*(1.28, 32.07) = 0.43, *p* = .57, η_p_^2^ = .02. There was no main effect of condition, *F*(2, 25) = 0.99, *p* = .39, η_p_^2^ = .07. See Fig 1 for an illustration of the produced mean time estimates per condition and see Fig 2 for an illustration of the produced mean time estimates per repeated measure and per condition.

The correlation analyses of the background variables (i.e., the results from the STAI, the SCL-90, and the S-ZTPI) and the produced mean time estimates of all participants yielded no significant correlations (see S1 Table). For a discussion of the results see “Discussion and conclusion”.

## Experiment 2

The aim of Experiment 2 was to investigate how an interaction between three conditions of video playback-speed, that is, slow (80%), normal (100%), and fast; (120%) and two levels of fps (i.e., 25 fps and 50 fps), during the depiction of observable motion, influences the production of intervals of time. The hypotheses were: a) that the slow playback-speed when using either high or low fps would result in overproductions of intervals of time compared with the normal playback-speed, b) that the fast playback-speed when using either high or low fps would result in underproductions of intervals of time compared with the normal playback-speed, c) that the slow playback-speed of 25 fps would give rise to smaller effects of overproduction of intervals of time compared with the slow playback-speed of 50 fps, d) that a fast playback-speed of 25 fps would result in a greater underproduction of intervals of time compared with the fast playback-speed of 50 fps. The predictions were in line with the background literature of Experiment 1 and the assumption that high and low fps correspondingly result in smooth and uneven motion [29]. The difference in the perception of motion was thought to lead, in accordance with an interpretation of Fraisse [23], to the CNS interpreting the uneven motion as more noticeable changes of events and, therefore, as faster motion, which in turn would lead to an increase of the effects of underproduction.

### Method

#### Participants

A convenience sample of 60 Swedish-speaking Finnish students (15 men and 45 women: age range 19 to 29) at Åbo Akademi University participated in the experiment. The participants were recruited via e-mail or were asked to join whilst visiting the university canteen. After selection, they were randomized into six separate experimental conditions (see Experiment 2: Procedure). All the participants gave their informed consent before taking part in the experiment. After completion, five cinema tickets were randomly allotted to five separate individuals (one ticket each) that took part in the experiment.

#### Measures

Prior to the experiment, a brief self-assessment form was administered in order to investigate factors shown to influence time production [7]. Experiment 2 differed from Experiment 1 in that only a single and much simpler questionnaire was employed consisting of a Likert scale ranging from one to five in order to measure three variables: Hunger, Tiredness, and State of Mind. The down-scaled questionnaire was deemed adequate considering the time-consuming nature of the questionnaires in Experiment 1 that did not yield any significant results (see Experiment 1: Results). In the questionnaire used in Experiment 2, the lower value “1” on the scale was defined as: very tired, very hungry, or very sad, and the upper value “5” on the scale was defined as: very awake, very satisfied, or very happy. Afterwards, participants were also asked to verbally account for any strategy that they employed in order to estimate time during the experiment. This was done in order to acquire exploratory qualitative data on chronometric strategies and was not part of the statistical analyses. The great majority replied that they counted, whereas a few said they, for example, visualized a clock ticking.

#### Stimuli

The stimuli used in Experiment 2 were created from a video sequence that was captured using a smart phone (Samsung Galaxy Alpha: SM-G850F) with a recording speed and standard playback-speed of 60 fps and a resolution of 1920 x 1080 pixels. The video was captured from the second floor of an indoor market space (i.e., the camera angle was filmed from above and tilted slightly downwards). The same indoor market space was used for both Experiment 1 and 2 but different angles were used. The video sequence was converted with the use of a computer software program (Sony Vegas Pro 13) to create two video sequences with the same duration (eight minutes) and the same content but with a different number of frames. The first video sequence was converted from 60 fps to 25 fps (resolution 1080 x 720) and the second sequence was converted from 60 fps to 50 fps (resolution 1080 x 720). In other words, the two video sequences (25 fps and 50 fps) displayed the exact same sequence of events over the exact same duration of time, but the number of frames in 25 fps video sequence was roughly half that of the frames in the 50 fps video sequence. Furthermore, as in Experiment 1, due to the manipulation of speed, there was a difference in the amount of content viewed per condition; that is, those in the fast condition saw more overall content of the same video sequence compared with those in the slow condition (see Table 1). The two video sequences were then used to create six conditions (see Experiment 2: Procedure) with the help of the software Presentation (version 12.1.) and were presented on a 2.8 GHz iMac computer with a 24-inch LED-monitor with a video playback display resolution of 800 x 1280 pixels.

**Table 1:** Duration and frames presented per condition in each of the video sequences used in Experiment 2.

FPS	Condition	Duration (sec.)	Frames presented
25	Fast	480.02	14401
	Normal	480.04	12001
	Slow	480.03	9601
50	Fast	481.43	28801
	Normal	480.03	24001
	Slow	480.02	19201

#### Procedure

The investigational method of Experiment 2 was largely identical to that of Experiment 1 when gathering observations, but Experiment 2 differed from Experiment 1 in four ways: 1) the number of participants was greater, 2) the video quality was higher 3) there was an additional manipulation of the number of frames used to depict motion, and 4) the length of the video playback was shorter. The six conditions used in Experiment 2 were as follows: 1) 25 fps at a slow speed, 2) 25 fps at a normal speed 3) 25 fps at a fast speed, 4) 50 fps at a slow speed, 5) 50 fps at a normal speed, and 6) 50 fps at a fast speed (Table 1). A total of 60 participants were randomly assigned into the six conditions (10 participants per condition) where they watched an eight minute long high-resolution video sequence. The choice of a shorter video sequence duration in Experiment 2 (i.e., eight minutes compared with 12 minutes in Experiment 1) was based on the wish to circumvent possible effects of tiredness due to prolonged exposure to the monotonous stimulus. Nevertheless, it was deemed necessary to keep the duration relatively high in order to retrieve as much information as possible during the experimental procedure. The eight minute duration was decided to be an adequate shortening of duration from the previous experiment. There is no evidence to support the notion that this difference in duration might impair or skew the results.

### Statistical analyses

The methods and motivations used for the statistical analyses of the data in Experiment 2 were almost identical to those outlined for Experiment 1 (see Experiment 1: Statistical analyses). However, the objectives of the repeated measures ANOVA in Experiment 2 was to investigate the main effects of condition and fps, as well as their interactive effect, on time production. Prior to the main analyses we found a total of 130 outliers that were removed from the dataset. Before removal there was a total of 3020 produced estimates and after removal there was a total of 2890 produced estimates. Additionally, due to the use of an eight minute long video sequence in Experiment 2, and not a 12 minute sequence as in Experiment 1, the three time clusters that were calculated per individual corresponded to 1) estimate 1 (the mean value of all estimates between 0–160 seconds), 2) estimate 2 (the mean value of all estimates between 161–320 seconds), and 3) estimate 3 (the mean value of all estimates between 321–480 seconds). Furthermore, prior to the main analysis the data was found to be normally distributed on each level of the independent variables, but three participants had to be removed due to the detection of outliers for two participants (the same boxplot analysis as in Experiment 1) and missing values for one participant in the clustered data distributions: two from the 25 fps fast condition and one from the 50fps fast condition. This resulted in 57 participants being included in the main analysis; leaving 10 participants in all conditions except for the fast 25 fps condition (8 participants) and the fast 50 fps condition (9 participants).

Furthermore, all background variables (i.e., hunger, tiredness, and state of mind) were correlated, using Pearson correlation (two-tailed), with the average produced time estimates in order to investigate whether or not there were any associations between the background data and the outcome data.

### Results

**Fig 3:**
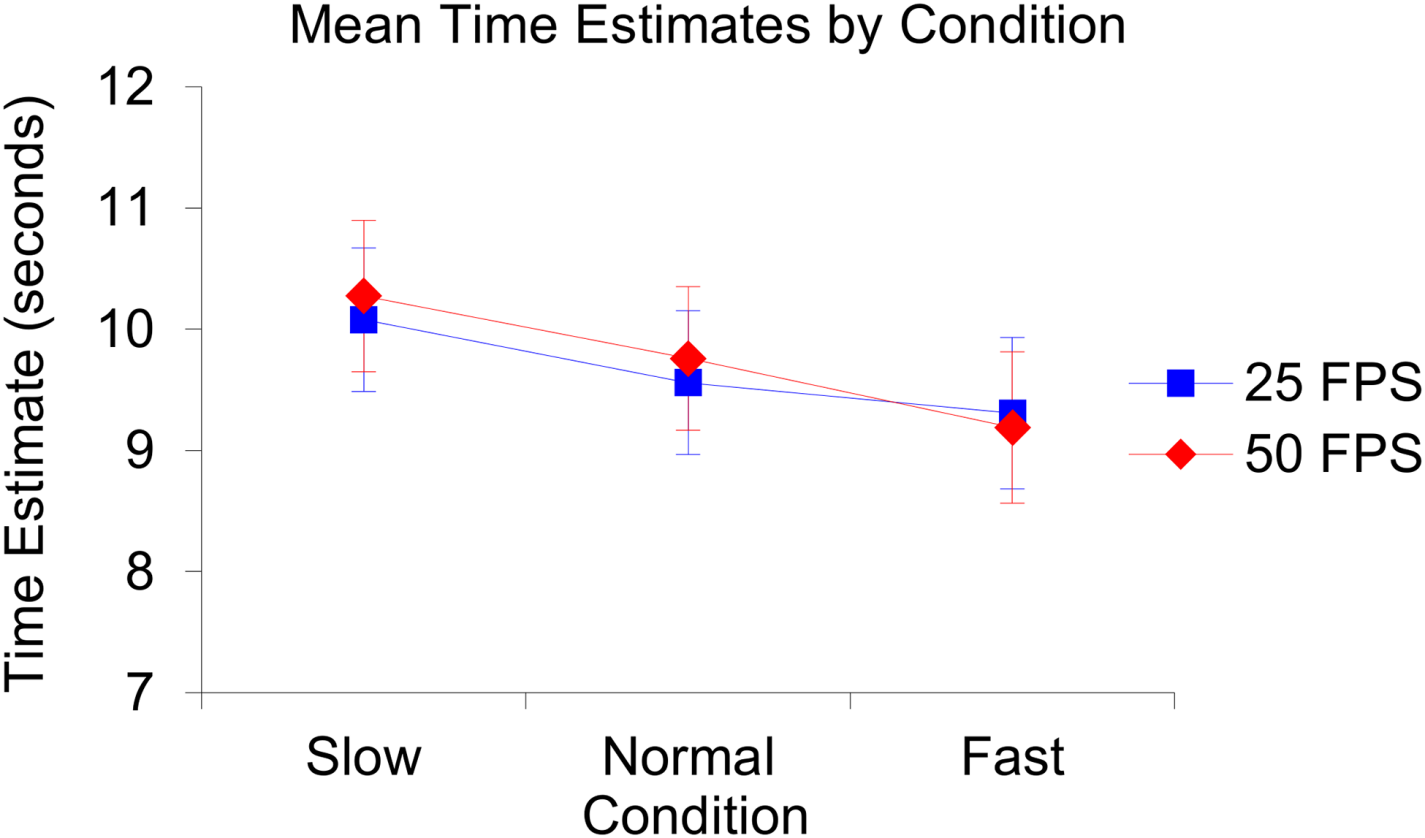
Illustration of the produced mean time estimates per condition and frame rate (fps). The slow 25 fps condition (*n* = 10, *M* = 10.08, *SE* = 0.59), the slow 50 fps condition (*n* = 10, *M* = 10.27, *SE* = 0.63), the normal 25 fps condition (*n* = 10*M* = 9.56, *SE* = 0.59), the normal 50 fps condition (*n* = 10, *M* = 9.76, *SE* = 0.59), the fast 25 fps condition (*n* = 8, *M* = 9.31, *SE* = 0.63), the fast 50 fps condition (*n* = 9, *M* = 9.188, *SE* = 0.63). The error bars represent the standard errors of the mean in each condition.

**Fig 4:**
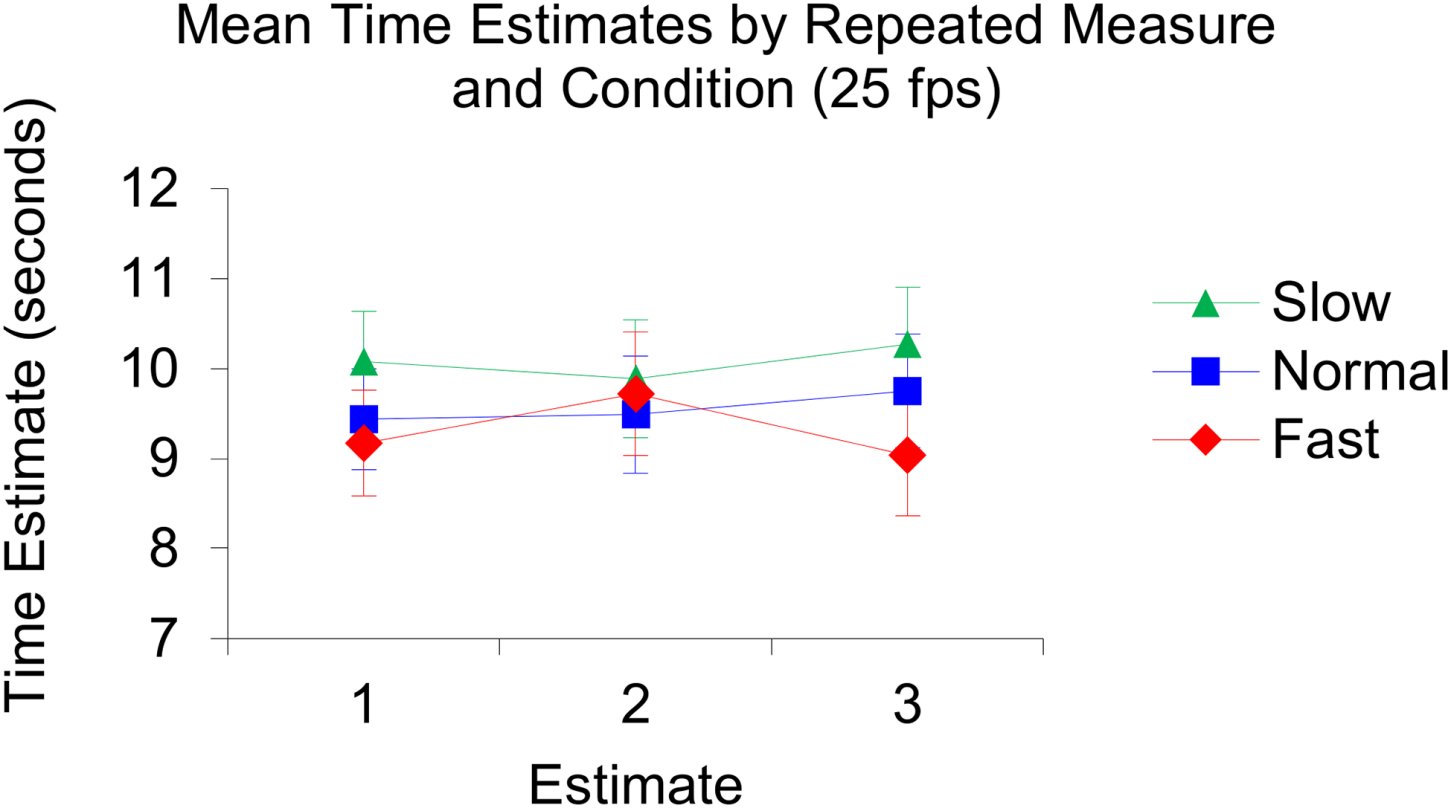
Illustration of the produced mean time estimates per repeated measure and per condition in the 25 fps setting. In the slow condition: Estimate 1; 10.08, estimate 2; 9.89, estimate 3; 10.27. In the normal condition: Estimate 1; 9.44, estimate 2; 9.49, estimate 3; 9.75. In the fast condition: Estimate 1; 9.17, estimate 2; 9.72, estimate 3; 9.03.

**Fig 5:**
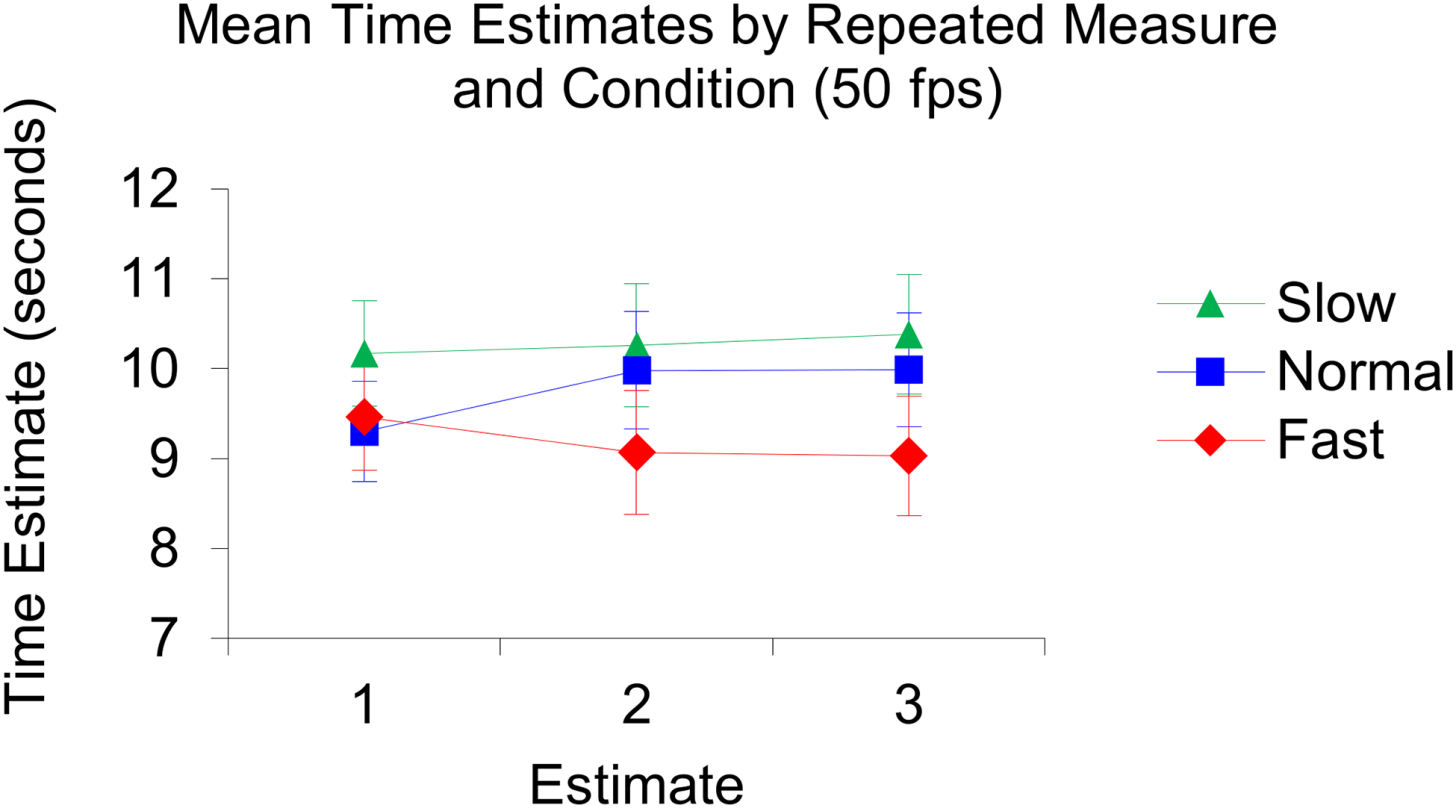
Illustration of the produced mean time estimates per repeated measure and per condition in the 50 fps setting. In the slow condition: Estimate 1; 10.17, estimate 2; 10.26, estimate 3; 10.39. In the normal condition: Estimate 1; 9.30, estimate 2; 9.98, estimate 3; 9.99. In the fast condition: Estimate 1; 9.46, estimate 2; 9.07, estimate 3; 9.03.

The repeated measures ANOVA revealed that the assumption of sphericity was not violated, (χ2(2) = 2.64, *p* = .29). The analysis revealed that there was no significant difference between the repeated measures, *F*(2, 102) = 0.82, *p* = .44, η_p_^2^ = .02. Analyses also revealed that there was no main effect of condition, *F*(2, 51) = 1.14, *p* = .33, η_p_^2^ = .04, no main effect of fps, *F*(1, 51) = 0.36, *p* = .85, η_p_^2^ < .01, and no interactive effect of condition and fps, *F*(2, 51) = 0.044, *p* = .96, η_p_^2^ < .01, on time production. Illustrations of the produced mean time estimates per condition and fps can be found in Fig 3. See also Fig 4 for an illustration of the produced mean time estimates per repeated measure and per condition in the 25 fps setting and Fig 5 for an illustration of the produced mean time estimates per repeated measure and per condition in the 50 fps setting.

The correlation analyses of the background variables (i.e., hunger, tiredness, and state of mind) yielded no significant results (see S2 Table). For a discussion of the results see “Discussion and conclusion”.

## Experiment 3

The aim of Experiment 3 was to bridge the gap between Experiments 1 and 2. The gap being that Experiment 1 used a video sequence that was filmed in much poorer quality compared to the sequence used in Experiment 2. Furthermore, neither Experiment 1 nor Experiment 2 included a baseline measure which could be used to investigate possible differences between groups prior to being randomly assigned to one of the three conditions and to investigate if time production differed during baseline and during experimental condition. The aims and hypotheses of Experiment 3 were identical to Experiment 1 (see Experiment 1).

### Method

#### Participants

A convenience sample of 30 Swedish-speaking Finnish students (including 18 women and 12 men; age range 17 to 47) at Åbo Akademi University participated in the experiment. Participants were recruited via e-mail or were verbally asked to join while visiting the university canteen. Participants were randomized into three separate experimental conditions as in Experiment 1 (see Experiment 1: Procedure). All participants gave their informed consent before taking part in the experiment. No compensation was given for participation.

#### Measures

In Experiment 3 the same pretest measures were used as in Experiment 2 (see Experiment 2: Measures). However, in contrast to Experiment 2, we also included the same measures at posttest in order to investigate if the test situation significantly altered hunger, tiredness or state of mind.

#### Stimuli

The stimulus video used in Experiment 3 was the same 25 fps sequences that was used in Experiment 2 (see Experiment 2: Stimuli) and the manipulation and duration of the video sequence was the same as in the 25 fps condition in Experiment 2 (see Experiment 2: Table 1). For the baseline measure, a single static frame was captured from the normal 25 fps video sequence. The static image was presented for three minutes during the baseline measure (see Experiment 3: Procedure).

#### Procedure

The procedure of Experiment 3 was the same as Experiment 1 except that i) a three minute baseline measure was included for all participants prior to being randomly assigned into one of three conditions and ii) the duration of the experiment was eight minutes (as in Experiment 2) instead of 12 minutes (as in Experiment 1). During the baseline measure the participants were asked to sit in front of a computer and continuously produce time intervals of nine seconds for 3 minutes (with exactly the same instructions as in all conditions of Experiments 1–3).

### Statistical analyses

The methods used for the main statistical analyses of the data in Experiment 3 were similar to those used in Experiment 1 (see Experiment 1: Statistical analyses). The differences were: i) that a preliminary one-way ANOVA was conducted on the produced time estimates during the baseline measure in order to ascertain if there were differences between groups. Also, ii) the inclusion of a baseline enabled three separate repeated measures ANOVAs to be conducted in order to investigate if there was a significant difference between the mean time productions in the baseline measure compared with the mean time production of each of the three conditions.

Initial investigation of the dataset revealed a total of 117 outliers that were consequently removed prior to analysis. Before removal there was a total of 2196 produced estimates and after removal there was a total of 2079 produced estimates. Additionally, due to the use of the same eight minute video sequence as was used in Experiment 2 the data was clustered exactly the same way as in Experiment 2 (see Experiment 2: Statistical analyses). Examination of the dataset after clustering revealed that the data was normally distributed on each level of the independent variable. Nevertheless, one participant was removed from the fast condition due to missing data after the data clustering; leaving a total of 29 participants (ten in both the slow and the normal condition and nine in the fast condition).

All background variables (i.e., hunger, tiredness, and state of mind) were correlated, using Pearson correlation (two-tailed), with the produced mean time estimates in order to investigate whether or not there was an association between the pretest data and the outcome data. Lastly, paired sample t-tests were conducted in order to investigate the difference between the pre- and posttest variables.

### Results

**Fig 6:**
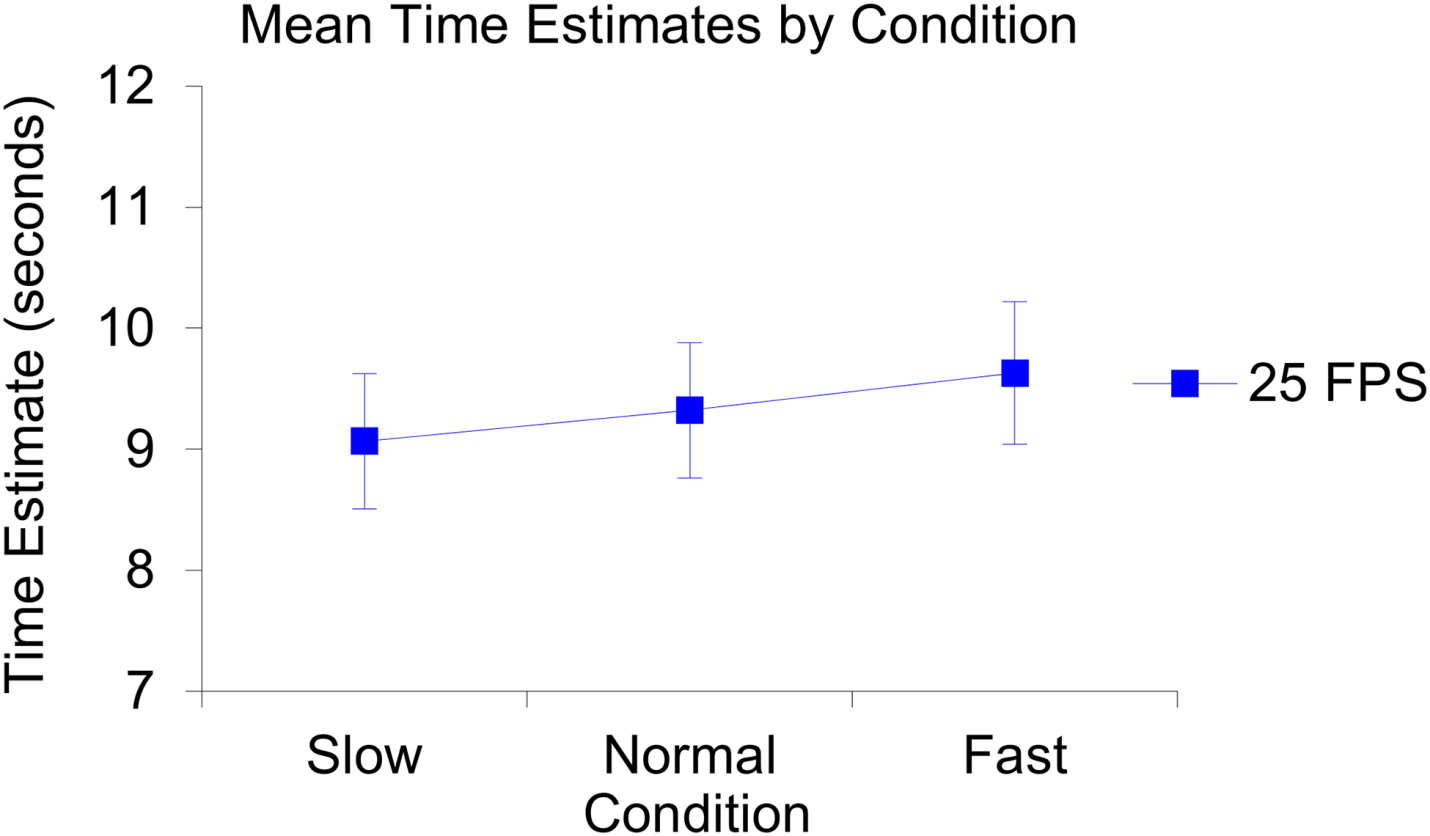
Illustration of the produced mean time estimates per condition. The slow condition *(n* = 10, *M* = 9.07, *SE* = 0.56), the normal condition *(n* = 10, *M* = 9.32, *SE* = 0.56) and the fast condition (*n* = 9, *M* = 9.63, *SE* = 0.59). The error bars represent the standard errors of the mean in each condition.

**Fig 7:**
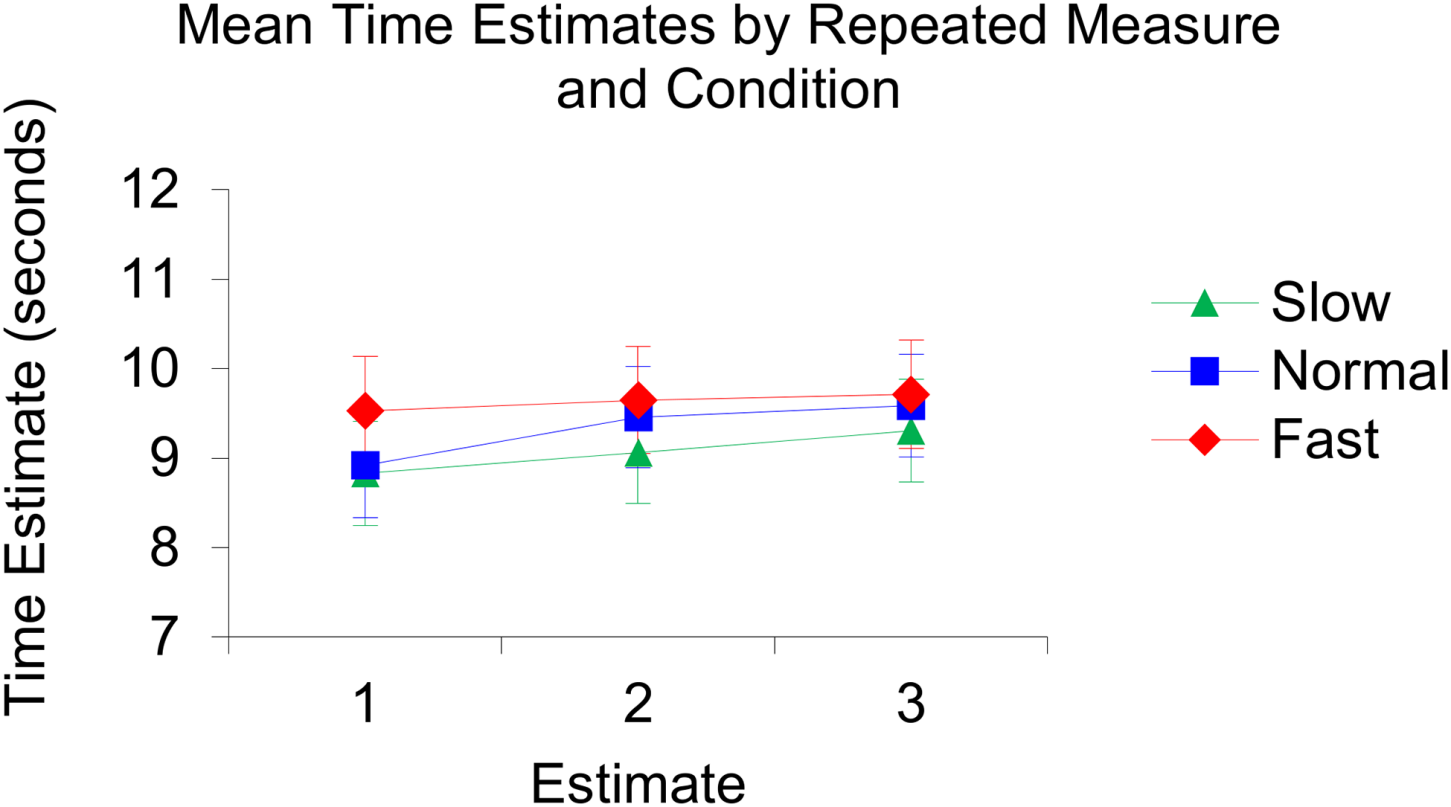
Illustration of the produced mean time estimates per repeated measure and per condition. In the slow condition: Estimate 1; 8.83, Estimate 2; 9.06, Estimate 3; 9.31. In the normal condition: Estimate 1; 8.92, Estimate 2; 9.46, Estimate 3; 9.59. In the fast condition: Estimate 1; 9.53, Estimate 2; 9.65, Estimate 3; 9.71.

The preliminary one-way ANOVA analysis investigating possible differences between groups in the baseline measure found no significant differences between groups, *F*(2, 28) = 0.06, *p* = .94. The repeated measures ANOVA investigating the effects of condition and produced time estimates showed that sphericity was not violated, (χ2(2) = 1.864, *p* = .39). There was a significant difference between the repeated measures, *F*(2, 52) = 5.54, *p* < .01, η_p_^2^ = .18, but there was no interactive effect of the repeated measures and condition, *F*(4, 52) = 0.68, *p* = .61, η_p_^2^ = .05, nor was there a main effect of condition, *F*(2, 26) = 0.24, *p* = .79, η_p_^2^ = .02. The repeated measures differed significantly between measures 1 and 3 (*M*_*diff*_ = -0.44, *SE* = 0.15, *p* = .02), but did not differ significantly between measures 1 and 2 (*M*_*diff*_ = -0.30, *SE* = 0.14, *p* = .13) or between measures 2 and 3 (*M*_*diff*_ = -0.15, *SE* = 0.18, *p* = .66). See Fig 6 for an illustration of the produced mean time estimates per condition and see Fig 7 for an illustration of the produced mean times estimates per repeated measure and per condition.

The significant difference between the repeated measures 1 and 3 indicated that the produced time estimates became shorter towards the end of the experiment across all conditions. Taking this into account, it was deemed prudent to conduct the three planned repeated measures ANOVAs (one per condition), that investigated the difference between time production in the baseline measure (static image) with time production in each of the three conditions (moving video sequence), by only including the first repeated measure of each condition. The first repeated measure (i.e., estimate 1) represented the produced mean time estimates per person for the first 160 seconds within a given condition. This was considered to be comparable to the produced mean estimates per person within the three minute (i.e., 180 seconds) baseline measure. The analyses from the three separate repeated measure ANOVAs revealed that there were no significant difference between produced time estimates in the baseline measure compared with the fast condition, *F*(1,9) = 0.19, *p* = .67, η_p_^2^ = 0.02, compared with the normal condition, *F*(1,9) = 1.56, *p* = .24, η_p_^2^ = 0.15, or compared with the slow condition, *F*(1,8) = 1.55, *p* = .25, η_p_^2^ = 0.16.

The correlation analyses of the pre-test variables (i.e., hunger, tiredness, and state of mind) and mean time production yielded no significant results (see S3 Table). The paired sample t-tests, investigating the difference between the pre- and posttest variables, yielded no significant difference for pretest (*M* = 2.79, *SD* = 0.74) and posttest (*M* = 2.64, *SD* = 0.78) measures of tiredness; *t*(27) = 1.44, *p* = .16, no significant difference for pretest (*M* = 3.25, *SD* = 0.93) and posttest (*M* = 3.18, *SD* = 0.95) measures of hunger; *t*(27) = 1.44, *p* = .16, but there was a significant difference for pretest (*M* = 3.61, *SD* = 0.69) and posttest (*M* = 3.46, *SD* = 0.58) measures of state of mind; *t*(27) = 2.12, *p* = .04. For a discussion of the results see “Discussion and conclusion”.

## Additional analyses

### Statistical analyses

Due to the lack of significant results in all three experiments, contrary to previous findings [14, 19], we deemed it logical to create a dataset based on the data from all three experiments and to conduct a one-way ANOVA in order determine if there was a main effect of condition on the production of time estimates. The dataset was compiled by using the exact same dataset as described in the three previous studies; that is, using the same number of produced time estimates and with the same outliers removed. Furthermore, considering that there was no main effect of fps or an interactive effect between fps and condition or between fps and the repeated measures in Experiment 2, we disregarded fps in the current analysis. This resulted in a dataset consisting of a total of 114 participants; 35 in the fast condition, 40 in the normal condition, and 39 in the slow condition. The data was normally distributed.

Lastly, due to the non-significant results of all experiments, we ran a separate Bayesian repeated measures ANOVAs on all three experiments and a Bayesian one-way ANOVA on the pooled data, using the computer software JASP [40], in order to quantify the evidence for the null hypothesis in all experiments [41].

### Results

**Fig 8:**
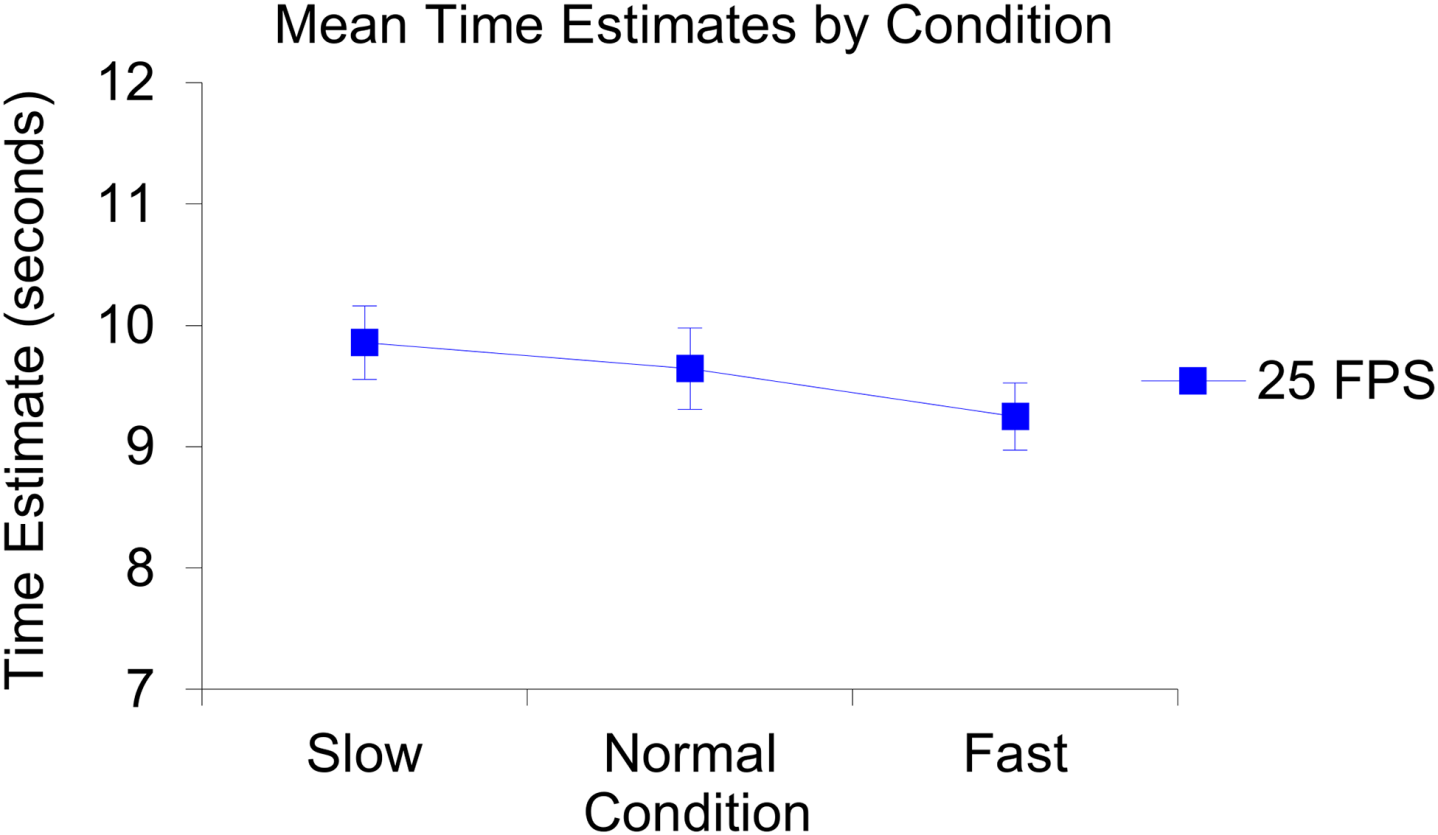
Illustration of the produced mean time estimates per condition. The slow condition *(n* = 39, *M* = 9.86, *SE* = 0.30), the normal condition *(n = 40*, *M* = 9.64, *SE* = 0.34) and the fast condition (*n* = 35, *M* = 9.25, *SE* = 0.27). The error bars represent the standard errors of the mean in each condition.

Results from the one-way ANOVA revealed no significant main effect of condition on time production, *F*(2, 113) = 0.96, *p* = .39. See Fig 8 an illustration of the produced mean time estimates per condition.

Results from the Bayesian repeated measures ANOVA yielded the following: Experiment 1: Condition *BF*_*01*_ = 1.55, Experiment 2: Condition *BF*_*01*_ = 2.05, fps *BF*_*01*_ = 3.88 and the interaction between condition and fps *BF*_*01*_ = 4.14, Experiment 3; Condition *BF*_*01*_ = 1.72. Results from the Bayesian one-way ANOVA for the pooled data were: Condition *BF*_*01*_ = 5.53. This analysis revealed that there was more support for the null hypothesis than for the alternative hypothesis; the alternative hypothesis being that video playback-speed has an effect on time production. This interpretation of the Bayes factor adheres to the rule that a Bayes factor *BF*_*01*_ (i.e., support for H0 over H1) above 1 is considered weak support for hypothesis one [41].

## Discussion and conclusion

### Study aims and hypotheses

The aims of the three experiments (Experiment 1, *n* = 28; Experiment 2, *n* = 57, Experiment 3, *n* = 29) included in this study were to investigate the effect of observed motion-speed through video playback on time production by manipulating, in Experiment 1 and 3, three conditions of video playback-speed, that is, slow (80%), normal (100%), and fast (120%), and, in Experiment 2, three conditions of speed (i.e., slow, normal, fast) and two kinds of fps (i.e., 25 fps and 50 fps). The hypotheses of Experiment 1 and Experiment 3(see Experiment 1) were based on findings from previous studies showing that slow and fast video playback-speeds respectively result in an over- and underestimations of time [14, 19]. The postulates of Experiment 2 (see Experiment 2**)** were deduced from i) the same background information as Experiment 1 concerning the effect of playback-speed on time production, ii) the suggestion that event changes play a central role in the CNSs assessment of time [23] so that more event changes are interpreted as an indication that more time has passed, iii) the fact that a high versus a low frame rate results in a smooth versus an uneven depiction of motion [29]. In Experiment 2, it was hypothesized that uneven motion would be perceived as containing more noticeable event changes compared with smooth motion and so the uneven motion of a low fps stimulus was thought to influence time production so as to result in greater underproductions of time compared with the influence of a high fps stimulus.

### Results and interpretations

Data analyses revealed no significant main effect of condition on time production in any of the three experiments, nor was there a main effect of fps or an interactive effect between condition and fps on time production in Experiment 2. Additionally, analyses revealed no significant correlations between pretest measures and time production in Experiment 1 (i.e., tiredness, anxiety, time orientation, and psychopathology), Experiment 2 (i.e., tiredness, state of mind, and hunger), or in Experiment 3 (i.e., tiredness, state of mind, and hunger). Analyses of the differences between pre- and posttest measures in Experiment 3 did, however, reveal a significant difference between pretest state of mind and posttest state of mind; *t*(27) = 2.12, *p* = .04. Nevertheless, the difference in pre- and posttest means were small (pretest; *M* = 3.61, *SD* = .69, posttest; *M* = 3.46, *SD* = .58) and since there was no significant correlation between produced time estimates and state of mind, the difference should perhaps not be interpreted as having relevance for the interpretations of the main results. Interestingly, analyses of the dataset of Experiment 3 revealed a significant difference between repeated measures 1 and 3, but not between measures 1 and 2 or between measures 2 and 3. The direction of the difference indicated that participants tended to produce shorter time estimates towards the end of the experiment. This could perhaps be interpreted as a result of impatience or of estimation becoming shorter due to repetitious nature of the task. However, similar results were not found in Experiment 1 or Experiment 2 (Experiment 2 also consisting of a much larger sample), which makes the result inconclusive. Additionally, it is worth mentioning that in Experiment 3 time production did not follow the same overall pattern as in Experiments 1 and 2; that is, in Experiment 3 the slow condition resulted in the shortest time productions in contrast to Experiments 1 and 2 where the slow condition resulted in longest time productions. Lastly, having pooled the data from all three experiments data analysis revealed no significant main effect of condition on time production.

The lack of a significant effect of playback-speed on the time production task in all three experiments was contrary to previous findings [14, 19]. However, the present study differed from previous studies in that we used a production task where participants continuously produced nine-second time estimates during video playback, instead of producing only one 20-second time estimate after video playback [19] or comparing the duration of two brief flashes presented during the video[14]. Importantly, in comparison with Grivel et al. [19], which included 16 participants, and Eagleman [14], which did not report a sample size, the present study included a relatively large sample size; that is 28 participants in Experiment 1, 57 participants in Experiment 2, and 29 participants in Experiment 3. Moreover, the present study employed a more subtle manipulation of playback-speed compared with previous studies [19]. Grivel et al. [19] used a manipulation of 50% playback-speed (slow condition) and 150% playback-speed (fast condition); whereas in the current study we used a manipulation of 80% (slow) and 120% (fast) playback-speed. The more subtle manipulation of playback-speed could be a reason for the different findings; that is, using a more drastic manipulation of playback-speed might be required in order to produce a noticeable effect on time production. It is also noteworthy that the general direction of Experiment 1 and Experiment 2, as well as the direction of the pooled data from all three experiments, follow the pattern of previous findings [14, 19]; that is that slow video playback tends to result in overproductions of intervals of time whereas fast playback tends to lead to underproductions of intervals of time. Taking this into account, it is nevertheless important to note that the results from Experiment 3 were the complete reverse of those found in Experiment 1 and 2, and none of the results in any of the experiments were significant. As to why the observed effect in Experiment 3 was reversed, we can only speculate. One plausible explanation is that the preliminary baseline measurement impacted the main experiment, through reactivity and/or chronometric strategy development, resulting in reduced overall variance. In this scenario, it is possible that the unexpected observed effect is a product of error variance.

### Limitations

The novelty of the current investigation of the effect of observed motion-speed during video playback on time production was that it examined not only the effects of motion-speed (slow, normal, and fast) but also the effects of fps (25 fps and 50 fps) and the interaction between fps and motion-speed, on the production of intervals of time. Understanding how fps and motion-speed interactively influence the visual observation of motion and how this affects temporal processing is a crucial step in understanding how motion in the real world influences time estimation and time production. However, future directions should take into account the limitations of the present study, which are that the manipulation of playback-speed in all three experiments was only 20% slower and 20% faster compared to normal playback-speed. It may be that such a subtle manipulation does not give rise to effects found by previous studies with more accentuated manipulations of playback-speed [19]. Furthermore, the continuous time production task might not be the best suited for such an investigation and perhaps a less repetitive task, such as a comparison task [14], could be used to better isolate the effects of video playback-speed on psychological time.

### Conclusion

In conclusion, the results from the present study are an important addition to the field of research aiming to investigate the effects of observed motion on time production. This is especially true because investigations into the effect of motion-speed on time production are commonly conducted with the aid of computer-simulated stimuli in research laboratories; using frame rates and playback-speeds to depict motion. Moreover, considering the varied use of different frame rates and speeds when depicting visual stimuli and movement in video games, cinematography, and virtual reality simulators, it is surprising that so little is known concerning the effects of different amounts of fps and playback-speeds on time production. The results from the present study indicate that subtle manipulations of video playback-speed or using higher or lower frame rates to record video sequences do not significantly impact time production. Future studies should instead aim to investigate the effects of more accentuated manipulations of video playback-speed on the production of time estimates. It is possible that there would be an interactive effect between frame rates and playback speed on time production if both frame rates and playback speeds were manipulated more drastically; based on the hypothesis that subjective time can be indexed by the perception of event changes.

## Supporting information

S1 File (PDF)

S1 Table (PDF)

S2 Table (PDF)

S3 Table (PDF)
