# Supplementary material for: As time passes by: Observed motion-speed and psychological time during video playback
Source: PLoS One. 2017 Jun 14;12(6):e0177855. doi: 10.1371/journal.pone.0177855 (PMC5470665; doi:10.1371/journal.pone.0177855)
Supplement: S1 File — (PDF) [file pone.0177855.s001.pdf]

Supporting information

Pilot experiment

A pilot experiment was conducted in order to investigate whether it was possible to manipulate the production of time intervals within individuals. Additionally the use of an explicit strategy was investigated (i.e., counting versus not counting) in order to investigate its possible influence on the production task.

A convenience sample of 12 Swedish-speaking Finnish students and staff-members from the Department of Psychology and Logopedics at Åbo Akademi University partook in the experiment. All participants were asked to take part in three successive experimental conditions. The three separate experimental conditions were created by altering the playback-speed of a video sequence (the video sequence used was the same as in Experiment 1; see Experiment 1: Stimulus). For all participants, the first condition was a baseline condition with normal video playback (i.e., 100%). For half of the participants, the second condition was the slow video playback (i.e., 80%) and the third condition was fast video playback (i.e., 120%). For the other half of participants these two conditions appeared in a reversed order (see S1 Fig).

The task in each condition was to sit in a secluded room and watch three low-resolution video sequences, which were each 5.25 minutes long, and to continuously produce nine-second intervals of time. The production of intervals of time was accomplished by pressing a keyboard key every time the participant perceived that nine seconds had elapsed from their last key press. The three video sequences were played in succession without any significant pause in-between playback. Half of the participants were asked not to use an explicit strategy when producing time intervals, the other half were not given any instructions. Stimuli were presented on 2.8 GHz

iMac computers with 24-inch LED-monitors and controlled by the software Presentation 12.1.

The data from the pilot experiment was analyzed by using the same method for removing of outliers and clustering of the data as in Experiment 1(see Experiment 1: Statistical analyses). However, the pilot data was analyzed using a Generalized Estimations Equation (GEE). Furthermore, due to the fact that all participants partook in all conditions in the pilot experiment, the first condition, which was always the normal-speed condition, was treated as a baseline condition and was removed from the analysis. That is, only the slow and fast conditions were used to analyze the data. The results from the pilot showed that there was a significant main effect of condition (slow vs. fast) (Wald χ^2 [1] = 174.39, $p < .001$) and of position (second vs. third; Wald χ^2 [1] = 189.29, $p < .001$). However, there appeared to be irreversible carry over effects; so that the effect of the first condition in position two dominated over the effect of the second condition in position three (see S1 Fig). Thus, we concluded that it was impossible to use a within-subjects design.

To test whether the manipulation had been successful, a second GEE analysis was conducted targeting only position two (outlined by the rectangle marked “A” in S1 Fig) and, thus, removing the contaminated condition in position three. The results approached significance, but were not significant (Wald χ^2 [1] = 3.29, $p = .070$). Nevertheless, the results were interpreted as adequate to further investigate the effects of playback-speed on time production, but by focusing on only one condition per individual so as to avoid order effects.

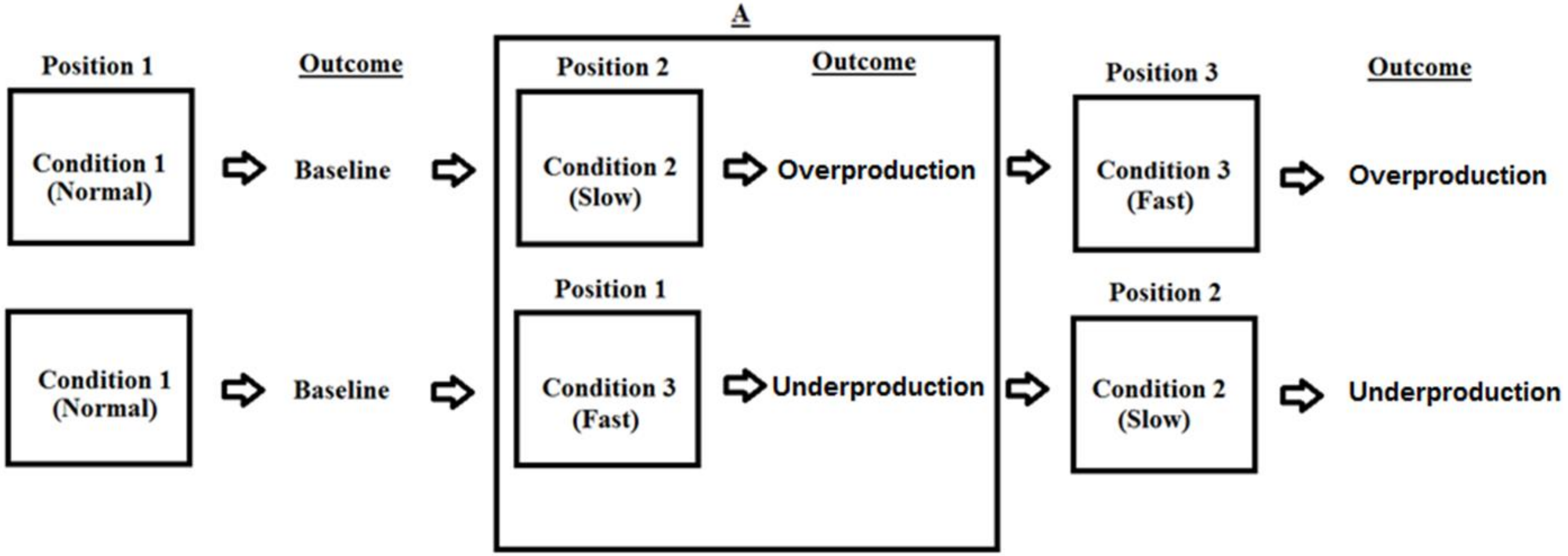

S1 Fig. Illustration of the experimental design and outcomes of the pilot experiment. Position one always contained normal playback-speed, whereas position two and three alternated between slow and fast playback speed. This was done in order to find out if there was an order effect when using first slow and then fast playback speed or using first fast and then slow playback-speed. The rectangle marked with the letter “A” represents the analysis conducted on the effects of only slow or fast condition on time production.
