# Supplementary material for: As time passes by: Observed motion-speed and psychological time during video playback
Source: PLoS One. 2017 Jun 14;12(6):e0177855. doi: 10.1371/journal.pone.0177855 (PMC5470665; doi:10.1371/journal.pone.0177855)
Supplement: S1 Table — (PDF) [file pone.0177855.s002.pdf]

# Supporting information

S1 Table. **Results from the Correlation Analyses of the Background Variables and the Overall Individual Mean of Time Production in Experiment 1**

| | Time Production (*M*) | |
|---|---|---|
| | Pearson Correlation | Sig. (2-tailed) |
| Symptom Checklist 90 | | |
| Global Severity Index | -.13 | .51 |
| Positive Symptom Distress Index | .00 | .93 |
| Positive Symptom Total | -.02 | .93 |
| Phobic Anxiety | -.27 | .17 |
| Obsessive- Compulsive | -.08 | .67 |
| Interpersonal Sensitivity | .074 | .71 |
| Depression | -.22 | .27 |
| Anxiety | .04 | .84 |
| Hostility/Anger | -.07 | .73 |
| Somatization | .09 | .67 |
| Paranoid Ideation | .14 | .48 |
| Psychoticism | -.04 | .83 |
| Zimbardo Time Perspective Inventory | | |
| Future Orientation | .15 | .46 |
| Present Fatalistic Orientation | -.02 | .90 |
| Present Hedonistic Orientation | -.20 | .31 |
| Past Positive Orientation | -.07 | .74 |
| Past Negative Orientation | .04 | .84 |
| State Trait Anxiety Inventory | | |
| STAI total Score | .20 | .30 |
| STAI total Score (Pre-test) | .01 | .63 |
| STAI total Score (Post-test) | .24 | .22 |

*Note.* N = 28. The Time Production (*M*) represents the mean production per individual.
