# Supplementary material for: As time passes by: Observed motion-speed and psychological time during video playback
Source: PLoS One. 2017 Jun 14;12(6):e0177855. doi: 10.1371/journal.pone.0177855 (PMC5470665; doi:10.1371/journal.pone.0177855)
Supplement: S2 Table — (PDF) [file pone.0177855.s003.pdf]

Supporting information

S2 Table. **Results from the Correlation Analyses of the Background Variables and the Overall Individual Mean of Time Production in Experiment 2.**

	Time Production (*M*)	
	Pearson Correlation	Sig. (2-tailed)
State of Mind	.01	.95
Tiredness	.00	.98
Hunger	-.11	.38

Note. N = 57. The Time Production (*M*) represents the mean production per individual.
