# Supplementary material for: As time passes by: Observed motion-speed and psychological time during video playback
Source: PLoS One. 2017 Jun 14;12(6):e0177855. doi: 10.1371/journal.pone.0177855 (PMC5470665; doi:10.1371/journal.pone.0177855)
Supplement: S3 Table — (PDF) [file pone.0177855.s004.pdf]

Supporting information

S3 Table. **Results from the Correlation Analyses of the Background Variables and the Overall Individual Mean of Time Production in Experiment 3**

	Time Production (*M*)	
	Pearson Correlation	Sig. (2-tailed)
Baseline measure - Mean Time Production		
State of Mind	-.06	.75
Tiredness	.18	.36
Hunger	-.12	.55
Condition - Mean Time Production		
State of Mind	-.12	.54
Tiredness	.17	.38
Hunger	-.12	.54

Note. N = 29. The Time Production (*M*) represents the mean production per individual.
